# The Use of Osteo-Inductive 3D-Printed Scaffolds Covered with a Pleiotrophin Peptide for Bone Defects: An In Vivo Experimental Study

**DOI:** 10.3390/bioengineering13060608

**Published:** 2026-05-24

**Authors:** Dimitrios Tsoumanis, Emilios E. Pakos, Ioannis Gkiatas, Ioannis Gelalis, Anna Batistatou, Evangelia Lampri, Despoina Deligianni, Evangelia Papadimitriou, Dimitrios Fotiadis, Anastasios Korompilias

**Affiliations:** 1 Department of Orthopaedics, University Hospital of Ioannina, 45110 Ioannina, Greece; epakos@yahoo.gr (E.E.P.); igkiatas@uoi.gr (I.G.); idgelalis@gmail.com (I.G.); koroban1960@gmail.com (A.K.); 2 Pathological Anatomy, University Hospital of Ioannina, 45110 Ioannina, Greece; abatista@uoi.gr (A.B.); elampri@uoi.gr (E.L.); 3 Department of Mechanical Engineering and Aeronautics, University of Patras, 26504 Patras, Greece; deligian@mech.upatras.gr; 4 Department of Pharmacy, University of Patras, 26504 Patras, Greece; epapad@upatras.gr; 5 Unit of Medical Technology and Intelligent Information Systems, University of Ioannina, 45110 Ioannina, Greece; fotiadis@uoi.gr

**Keywords:** bone defects, bone healing, scaffolds, osteoinduction, angiogenesis

## Abstract

The present study investigated the effect of a 3D-printed nanocomposite scaffold on bone healing in vivo. The scaffolds used were made from the bioresorbable thermoplastic polycaprolactone polymer, blended with Multi-Walled Carbon Nanotubes functionalized with chitosan, and manufactured with a rectilinear infill pattern and interconnected pores of 500 μm in size. The study included three groups of 10 Wistar rats, in which a 2 mm bone defect was created in the middle of the right femur. In the scaffold/peptide group, the gap was filled with the scaffold loaded with a peptide corresponding to human pleiotrophin amino acids 48-56 (PTN_48-56_), and the fracture was stabilized with a 12 mm K-wire as an intramedullary nail. In the scaffold group, the scaffold did not contain the peptide, and in the control group, the bone defect was stabilized without the use of a scaffold. Radiological examination revealed that bone healing was achieved on average in 6.6 weeks in the scaffold/peptide group, 7.2 weeks in the scaffold group, and 8.1 weeks in the control group. Histopathological examination performed 2 weeks postoperatively showed that angiogenesis in the scaffold/peptide group was 1.5 times higher than in the scaffold group and 2.5 times higher than in the control group. In conclusion, our osteo-inductive 3D-printed scaffold covered with PTN_48-56_ is a promising option for accelerating bone defect healing.

## 1. Introduction

Bone defects are a major problem in orthopaedic surgery. They are characterized by loss of bone mass that cannot be spontaneously reversed. In orthopedics, a critical-size bone defect is the smallest lesion that cannot be healed on its own and requires intervention. Causes of a bone defect may include a fracture, tumor resection, infection, nonunion, and others. Treatment of bone defects, particularly severe ones, remains a subject of debate, partly due to the challenge of determining whether a defect qualifies as critical in size [1]. However, in the existing literature, there are some benchmarks about the crucial bone defects, such as the length of defect that exceeds 1 to 2 cm, a loss of over 50% of the bone’s circumference, or a bone defect that reaches 1.5 to 2 times the diameter of the bone [2]. The main treatment option for this condition is autologous bone grafting, which has been used for the last few decades [3,4]. Other options are the Ilizarov fixation system and the Masquelet techniques. However, concerns for all these techniques relate to several reasons. Grafting is not always applicable across all anatomic areas; the Ilizarov fixation system and the Masquelet techniques are demanding, require an extended treatment period, and carry a risk of pin-track infection [5,6]. Due to the difficulties in treating large bone defects, their treatment remains a surgical, socioeconomic, and investigative challenge [7]. Over the last few years, the evolution of 3D printing technology and the improved mimicking of bone microarchitecture and pore systems have led to increasing research interest in 3D-printed scaffolds for covering bone defects [8,9]. The ideal bioscaffold should exhibit excellent biocompatibility, minimal toxicity, low immunogenicity, appropriate porosity and pore size, adequate biomechanical strength, the capacity to promote cell or tissue differentiation, regenerative surface activity, and controlled biodegradability [10].

Bioscaffolds are often loaded with growth factors to enhance osteogenesis and ideally angiogenesis. Such factors include bone morphogenetic protein 2 (BMP2), platelet-derived growth factor, fibroblast growth factors (FGFs), vascular endothelial growth factor A (VEGFA), and others. Some of these factors have shown some success, e.g., BMP2, but numerous problems remain unresolved [11,12]. These are mostly related to the challenge of keeping them in the area long enough and controlling the release of factors from the scaffold, the lack of standardization in delivery methods, and the high cost [13]. Pleiotrophin (PTN) is a growth factor that is abundant during embryonic life but has restricted expression in adulthood. One of the initial names of PTN, when it was isolated from the murine MC3T3-E1 osteoblasts, was osteoblast-stimulating factor 1. PTN exerts complex effects on osteoblast function in vitro and, although the mechanisms underlying its role in bone regeneration remain unclear, it generally promotes bone formation [14,15,16,17]. Peptides are often favored over proteins in bone scaffolds because of their lower immunogenicity, smaller molecular size, ease of synthesis and modification, lower cost, greater stability, and enhanced resistance to degradation caused by temperature and pH variations [18]. To the best of our knowledge, this is the first in vivo study to evaluate a 3D-printed scaffold composed of polycaprolactone reinforced with multi-walled carbon nanotubes, functionalized with medium-molecular-weight chitosan, and coated with a PTN-derived peptide to simultaneously promote osteogenesis and angiogenesis during bone defect healing.

## 2. Materials and Methods

### 2.1. Scaffolds Preparation

**Figure 1:**
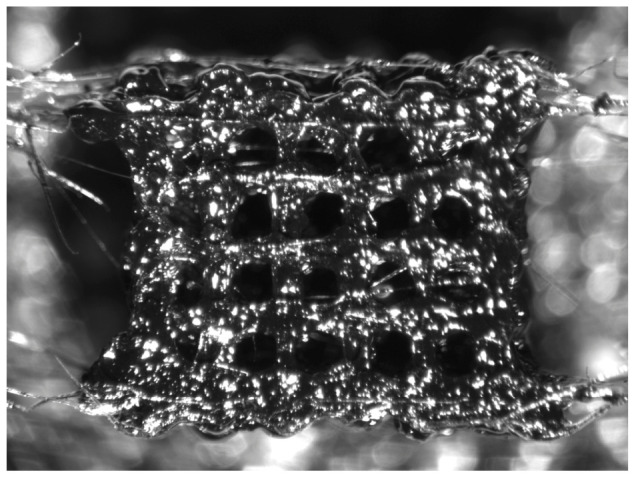
Reinforced PCL scaffold before compression. The scaffold used in this study was porous, featuring dimensions of 5 × 5 × 5 mm^3^ and a porosity of 45% [19].

The 3D-printed scaffolds used in the present study were made of polycaprolactone (PCL) reinforced with multi-wall (MW) carbon nanotubes (CNTs) and functionalized with medium-molecular-weight chitosan, as previously described (Figure 1) [19]. All scaffolds were sterilized under UV radiation for 20 min on each side. The scaffolds were then immersed in a PBS pH 7.4 solution containing the PTN_48-56_ peptide (derived from human PTN, UniProt accession number P21246) at 1 μg/mL at 4 °C for 24 h, allowing physisorption of the peptide onto the scaffolds. The peptide was synthesized and purified as previously described [20]. Uncoated scaffolds were immersed in PBS. Following this step, the scaffolds were carefully washed with sterile PBS and left to air dry at room temperature in a sterile environment until use. CNTs have been incorporated into 3D nanocomposite scaffolds to increase nanocomposite elasticity and to trigger cell differentiation when exposed during biodegradation. MWCNTs were purchased from Nanografi Nanotechnology (Ankara, Turkey), with specific properties (i.e., an outside diameter of 48–78 nm and purity of more than 96%). Medium-molecular-weight chitosan (CS) was purchased from Glentham (Corsham, UK). The PCL synthetic polymers, used as matrices, were purchased from Thermo Scientific (Waltham, MA, USA). To achieve the maximum filler content, while ensuring that the reinforced materials remained printable and did not negatively affect the initial mechanical properties, the MWCNTs were incorporated at a weight fraction of 40% in medium-molecular-weight CS (1,250,000 avg) in a CS/MWCNT product. Subsequently, PCL pellets were pulverized and thoroughly mixed with the CS/MWCNT product at a weight fraction of 5%.

### 2.2. Animal Handling

**Figure 2:**
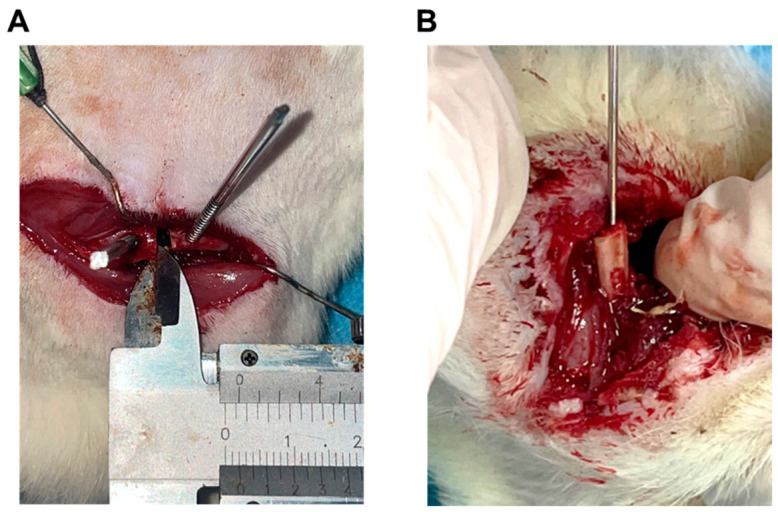
(**A**) The 2 mm bone defect. (**B**) Intramedullary nailing using a K-wire 12 mm.

Thirty male Wistar rats were bred at the Center for Animal Models of Disease at the University of Ioannina, Greece (EL33-BIObr01), under a controlled environment of 12 h light/dark cycles and food/water consumption ad libitum. The experiment was approved by the Directorate of Agricultural Economy & Veterinary Medicine of the Region of Epirus, carried out at the Laboratory of Microsurgery of the University of Ioannina (EL33-BIOexp02). The approximate weight of each rat was 350 g. The rats were divided into three groups. Each group contained 10 animals. All surgical procedures were carried out under general anesthesia by intraperitoneal injection of 75 mg/kg ketamine and 5–8 mg/kg xylazine. Tramadol (20 mg/kg) was used for analgesia and was administered via intraperitoneal injection during general anesthesia of the animals. In all cases, a lateral approach to the right femur was used to expose the bone under sterile conditions. In all rats, a bone defect of 2 mm was created in the middle of the right femur of each animal. In the scaffold/peptide group, the gap was filled with the 3D-printed scaffold loaded with PTN_48-56,_ and the fracture was stabilized with a 12 mm K-wire as an intramedullary nail. In the scaffold group, the scaffold was free from the peptide. In the control group, the bone defect was stabilized without a scaffold (Figure 2). Before wound closure, vancomycin was applied locally within the tissues for antibiotic prophylaxis, and penicillin was injected intramuscularly for 3 days in all animals. According to GPower 3.1, four animals from each group underwent radiological evaluation, and six were used for immunohistochemistry.

### 2.3. Radiological Evaluation

The radiological evaluation of the osteogenesis was performed with a portable high-frequency X-ray unit (meX+100 [PXP-100CA]) (medical ECONET GmbH, Oberhausen, Germany)and a Computed Radiography (CR) digitizer (Agfa CR 10-X). The animals were under general anesthesia during these procedures. Radiographic evaluations were performed at regular intervals (1st, 2nd, 4th, 6th, 8th week) to assess bone healing. Complete bone healing was defined as radiographic bridging of the defect with continuous mineralized callus formation across the cortical margins and disappearance of the fracture gap. Radiographic assessment was performed independently by two observers.

### 2.4. Histochemical and Immunohistochemical Analyses

Two weeks following the surgical procedure, the right femur from six rats per group was sent for immunohistochemical examination to evaluate angiogenesis. Rat specimens were fixed in 4% PBS-buffered paraformaldehyde for 72 h and then decalcified in 10% EDTA (pH 7.4) for 7 days at room temperature. After washing with water, the specimens were progressively dehydrated in ethanol solutions of increasing concentration (50–100% *v*/*v*), cleared in xylene, and embedded in paraffin wax. Serial sections were cut in the transverse plane at 4 μm using a microtome, and then stained with hematoxylin and eosin (H&E) or immunohistochemical stains. Immunostaining was performed with a DakoCytomation Autostainer Instrument (DakoCytomation, Kyoto, Japan). Briefly, 4 μm thick tissue sections were dewaxed in xylene and rehydrated in decreasing concentrations of ethanol. Endogenous peroxidase activity was blocked by incubation with peroxidase-blocking solution (DakoCytomation) for 5 min. Antigen retrieval was carried out by autoclaving the sections for 30 min in target retrieval solution (pH 6.0, DakoCytomation). The primary antibody used in the current study was against CD31 (clone JC/70A). Using an Envision Kit (Dako); slides were incubated with horseradish peroxidase-labeled polymer conjugated with secondary antibody for 30 min and then with substrate chromogen (diaminobenzidine) solution, followed by light counterstaining with Mayer’s hematoxylin. In each case, a control was used. Immunohistochemical evaluation of the differences among groups was assessed blindly. Photographs of the sections were taken under an Olympus BX43 microscope equipped with an Olympus SC30 digital camera (Olympus Optical, Tokyo, Japan). The percentage of positively stained cells or staining intensity was scored.

MVD scoring

The microvessel density (MVD) was determined by counting CD31-positive vessels. In each case, three areas with the highest microvascular density (so-called “hot spots”) were identified at ×100 magnification. Within each of these three areas, CD31-positive vessels were counted at ×400 magnification. The mean number of vessels across these three areas was calculated and considered as the MVD value for each case. Single endothelial cells and clusters of endothelial cells, with or without a visible lumen, were considered as individual vessels.

### 2.5. Statistical Analysis

Data are expressed as mean ± S.D., and one-way ANOVA was performed to compare values among the three experimental groups. A *p*-value < 0.05 was considered statistically significant. Statistical analysis was conducted using GraphPad Prism. All groups satisfied the Shapiro–Wilk normality test, and Tukey’s HSD (Honestly Significant Difference) test was applied for pairwise comparisons among all groups. Bullets in graphs represent different rats.

## 3. Results

### 3.1. Radiological Evaluation

**Figure 3:**
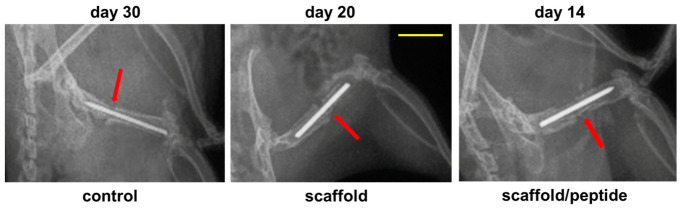
Representative radiographs were taken on the postoperative days when the callus was first visible in each group. Callus formation appears grayish (red arrows), while the compact bone appears more radiopaque. Control: no scaffold was used; Scaffold: an uncoated scaffold was used; Scaffold/peptide: a scaffold coated with the PTN_48-56_ peptide was used. All photos have the same magnification, and the yellow scale bar corresponds to 1 cm.

**Figure 4:**
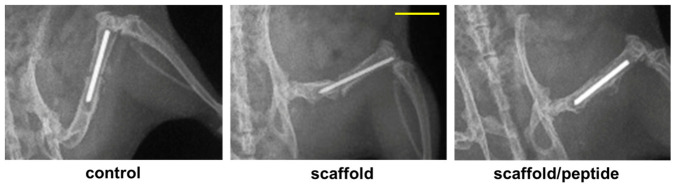
Representative radiographs 6 weeks postoperatively. Compact bone appears more radiopaque. Complete bone regeneration is observed only when the scaffold is coated with the PTN_48-56_ peptide. Control: incomplete bone healing when no scaffold was used; Scaffold: incomplete bone healing when an uncoated scaffold was used; Scaffold/peptide: successful bone healing when a scaffold coated with the PTN_48-56_ peptide was used. All photos have the same magnification, and the yellow scale bar corresponds to 1 cm.

**Figure 5:**
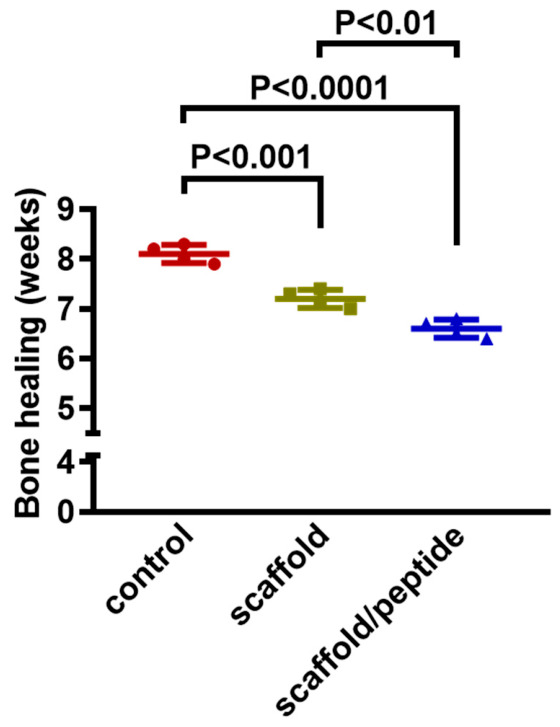
Duration for complete bone healing in weeks. Data are expressed as mean ± S.D. of the duration in weeks needed for complete bone healing. Control: no scaffold was used; Scaffold: an uncoated scaffold was used; Scaffold/peptide: a scaffold coated with the PTN_48-56_ peptide was used.

We observed bone defects and callus formation at 1st, 2nd, 4th, 6th, and 8th weeks after surgery. On radiographs, the callus seems to be grayish (more radiolucent), while the compact bone appears to be more radiopaque [21]. In the first week, radiographs revealed fractures with bone defects and no bone regeneration in all animals from all three groups. The callus was formed on day 14 in the group scaffold/peptide, on day 20 in the group scaffold, and on day 30 in the control group (Figure 3). The process of bone healing was continuously progressing (Figure 4), and callus formed into compact bone on average in 6.6 weeks in the scaffold/peptide group, 7.2 weeks in the scaffold group, and 8.1 weeks in the control group (Figure 5). Differences among groups regarding bone healing time were statistically significant (*p* < 0.05).

### 3.2. Immunohistochemical Evaluation

**Figure 6:**
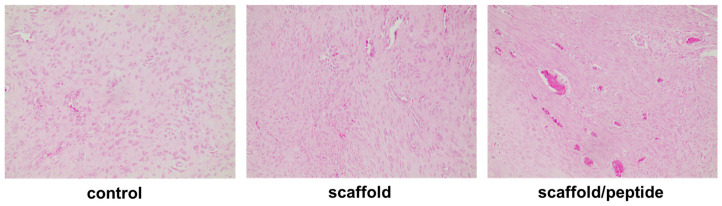
Hematoxylin and eosin (H&E)-stained sections at ×200 magnification demonstrating vascular structures in the three experimental groups: Control, in which no scaffold was used; Scaffold, in which an uncoated scaffold was applied; and Scaffold/peptide, in which a scaffold coated with the PTN_48-56_ peptide was used.

**Figure 7:**
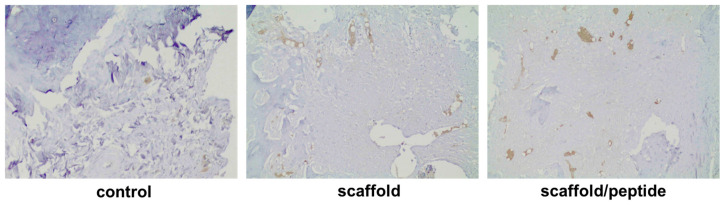
Representative photographs of new bone tissue sections at ×200 magnification stained for endothelial cells using a CD31-specific antibody (brown), two weeks postoperatively. Control: no scaffold was used; Scaffold: an uncoated scaffold was used; Scaffold/peptide: a scaffold coated with the PTN_48-56_ peptide was used.

**Figure 8:**
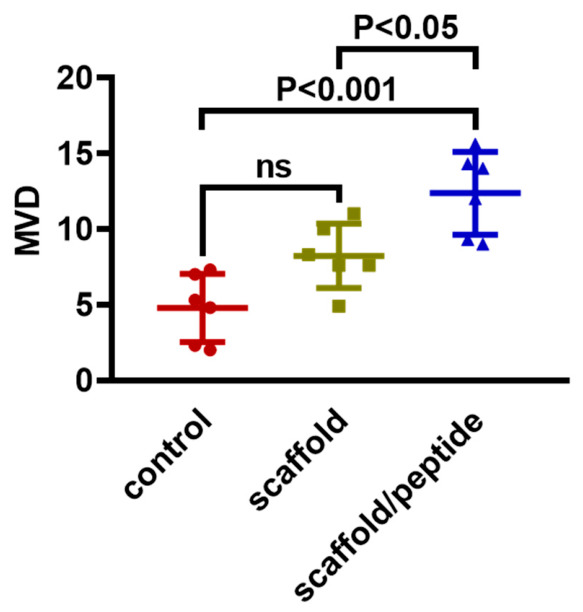
MVD two weeks postoperatively. Data are expressed as mean ± S.D. of the MVD. Control: no scaffold was used; Scaffold: an uncoated scaffold was used; Scaffold/peptide: a scaffold coated with the PTN_48-56_ peptide was used.

Angiogenesis was evaluated two weeks postoperatively by quantitative measurement of CD31-positive vessels. We observed that MVD was 1.5 times greater in the scaffold/peptide group than in the scaffold group and 2.5 times greater than in the control group (Figure 6, Figure 7 and Figure 8). These data suggest that although the uncoated scaffold also enhances angiogenesis, its coating with the PTN peptide has a much greater effect, aligning with its impact on bone healing.

## 4. Discussion

The present study presents a novel 3D-printed nanocomposite scaffold that enhances osteogenesis and angiogenesis in an in vivo model of bone healing. Our findings demonstrated that the scaffold/peptide construct resulted in earlier callus formation, faster radiographic union, and increased angiogenesis compared with both the unloaded scaffold and the control group. These results suggest that the combination of the osteoconductive scaffold with the PTN-derived peptide may synergistically promote bone regeneration and vascularization in vivo. Our efforts align with numerous similar approaches to develop scaffolds that accelerate bone healing [10,22,23,24], especially in fractures of delayed union or nonunion, which account for approximately 2–10% of fractures [25]. Over the past few decades, 3D-printed scaffolds have garnered significant attention because of their unique physical properties in tissue regeneration engineering, particularly for bone regeneration. This is due to their three-dimensional porous structure, desirable porosity, and favorable mechanical properties, which can mimic the natural trabecular bone [26]. The originality of the present study lies not only in the use of a 3D-printed nanocomposite scaffold, but also in the incorporation of the PTN_48-56_ peptide, which appears to enhance both osteogenesis and angiogenesis in vivo.

Our 3D-printed scaffold results in faster bone healing of the bone defect, even when used unloaded. Based on the radiographic examination, both callus formation and its maturation into compact bone occur more rapidly than in the absence of any scaffold, suggesting that our scaffold enhances osteogenesis in vivo. This may be due to the presence of CS on the scaffold. CS has been shown to maintain growth factors in active form and promote the differentiation of adipose-derived stem cells into osteoblasts through osteogenesis. Mesenchymal stem cells also undergo osteogenic differentiation in a scaffold consisting of hydroxyapatite, gelatin, and CS. At a wound site, a chemically tuned CS acts as an unsurpassable scaffold for the proliferation and differentiation of seeded stem cells [27,28,29].

In addition to osteogenesis, our scaffold also induces angiogenesis, a critical step in complete fracture healing. Deficient angiogenesis constitutes an inhibitory factor that leads to delayed union or nonunion [30] and insufficient or delayed fracture repair in the elderly [31]. It is now well accepted that the enhancement of vascularization concomitantly with osteoinduction is essential in the field of bone tissue engineering [32]. Nevertheless, simultaneously inducing these two biological processes remains challenging. CS has been found to enhance bone repair through both osteogenesis and angiogenesis [33] and may have contributed to the effect of our unloaded scaffold. However, the efficacy of CS is limited [33], in line with the significantly greater effect when we loaded the PTN peptide.

The most used growth factors for loading CS-containing scaffolds are VEGFA, bone morphogenetic protein 2 (BMP2), or parathyroid hormone (PTH) [33]. Loading of a scaffold with VEGFA would enhance angiogenesis but inhibit osteoblast activation [34,35]. BMP2 appears to stimulate both osteogenesis and angiogenesis in vivo [24]; however, studies in isolated endothelial cells indicate minimal to no effect on endothelial cell functions [36]. However, it significantly enhances the angiogenic effects of VEGFA or FGF2 [36], and this may explain why it stimulates angiogenesis when used on scaffolds in vivo to induce bone healing [35]. However, it has been shown that both VEGFA and BMP2 induce overexpression of inhibitor of DNA-binding 1 gene, and retard terminal differentiation of osteoblasts and bone formation [36]. PTH stimulates bone formation and seems to indirectly affect angiogenesis by increasing VEGFA release by osteoblasts [37]. On the other hand, locally applied PTH has osteoclastic and bone-resorptive effects [38], which hampers its use.

PTN is a growth factor that regulates both osteogenesis [14,17,39] and angiogenesis [40], and incorporated into mesoporous silica nanoparticles, it has been shown to potently induce osteoblast differentiation in vitro in the absence of an osteogenic differentiation-promoting medium [41]. When adsorbed onto three-dimensional biodegradable porous scaffolds, PTN exhibited osteogenic properties [42], supporting the notion that it can be used to stimulate bone formation. PTN is a highly conserved protein that interacts with numerous receptors through different domains [40]. In the present study, we used a peptide corresponding to amino acids 48-56 of the protein, which stimulates endothelial cell migration (Evangelia Papadimitriou, unpublished data). When the scaffolds were loaded with PTN_48-56_, osteogenesis was significantly accelerated, and angiogenesis was significantly enhanced compared with both the unloaded scaffold and the no-scaffold groups. Since PTN_48-56_ is a nine amino acid peptide, it is more stable than the PTN protein, making it easier, faster, and less expensive to acquire in large quantities for all potential applications, supporting its use over PTN or any other growth factor. More detailed studies are currently being conducted to investigate its effects on osteoblasts and endothelial cells, as well as their respective progenitor cells.

Our in vivo model is similar to previously used models for fracture healing. For example, Haffner-Luntzer et al. stabilized the fracture of the proximal femur with intramedullary nailing by introducing a 24-G needle [43]. Dong et al. employed 3D-printed PCL scaffolds to repair bone defects in rabbits measuring 6 mm in diameter and 4.5 mm in depth. They also observed that the new tissue entered the scaffold at 8 weeks, and the bone defect was invisible 12 weeks postoperatively [23]. Overall, the accelerated healing and increased angiogenesis observed in the present study support the potential translational value of the PTN_48-56_-coated scaffold for the treatment of bone defects. However, our study has some limitations. First, we selected intramedullary nailing to fix the fracture, which is not too rigid and may influence the outcomes. Our preferred option would be external fixation, but it was unable to maintain fracture reduction, and using a plate was difficult due to the small specimen size. Secondly, the evaluation of angiogenesis was performed by only measuring the MVD with the use of CD31 as an endothelial-specific marker. Although the abundance of blood vessels in the scaffold/peptide group are also evident in the H7E stained tissue sections, measurement of additional factors and markers would be beneficial towards a more complete histopathological assessment. In the present study, the release kinetics of PTN_48-56_ from the scaffold and the degradation profile of the scaffold in vivo were not evaluated. Future studies including detailed release and biodegradation analyses are warranted to further characterize the biological behavior of the scaffold system. Finally, we used only plain radiographs for the assessment of bone healing. A radiographic evaluation using micro-CT with determination of bone mineral density, bone tissue volume/total tissue volume, trabecular thickness and trabecular separation spacing would provide more detailed and objective information regarding the quality of the newly formed bone.

## 5. Conclusions

The proposed osteo-inductive 3D-printed scaffold enriched with the PTN_48-56_ peptide is a promising option for covering bone defects, enhancing both bone healing and angiogenesis. The fact that it is a 3D-printed scaffold also offers the advantage of customization. We also introduce a novel PTN-derived peptide that appears to couple osteogenesis and angiogenesis in vivo, highlighting the potential originality and translational value of this scaffold design.

## 6. Patents

These data have been included in a patent: Despoina Deligianni, Evangelia Papadimitriou, Vasilios Kostopoulos, Constantinos Athanassopoulos, Emilios Pakos, Anastasios Korompilias, Ioannis Gkiatas, Dimitrios Fotiadis, Maria Roubi, George Matsopoulos, Ioannis Kakkos. Biomimetic nanocomposite three-dimensional scaffolds for bone regeneration. Hellenic Industrial Property Organization Patent Application No. 20260100023/14.01.2026).

## Data Availability

Data that support the findings of this study are available from the corresponding author upon reasonable request.

## Abbreviations

The following abbreviations are used in this manuscript:

BMP-2	Bone Morphogenetic Protein 2
chitosan	CS
CNTS	Carbon Nanotubes
FGFs	Fibroblast Growth Factors
Multi-wall	MW
VEGFA	Vascular Endothelial Growth Factor A
PCL	Polycaprolactone
PTN	Pleiotrophin
MVD	Microvessel Density
